# Patients with Chronic Spinal Cord Injury Display a Progressive Alteration over the Years of the Activation Stages of the T Lymphocyte Compartment

**DOI:** 10.3390/ijms242417596

**Published:** 2023-12-18

**Authors:** Sergio Haro, Ana M. Gomez-Lahoz, Jorge Monserrat, Mar Atienza-Pérez, Oscar Fraile-Martinez, Miguel A. Ortega, Cielo García-Montero, David Díaz, Elisa Lopez-Dolado, Melchor Álvarez-Mon

**Affiliations:** 1 Department of Medicine and Medical Specialities, University of Alcala, Crta N-II km 33.6, 28871 Alcala de Henares, Spain; sergioharogiron@gmail.com (S.H.); alahoz1199@gmail.com (A.M.G.-L.); jorge.monserrat@uah.es (J.M.); oscarfra.7@hotmail.com (O.F.-M.); miguel.angel.ortega92@gmail.com (M.A.O.); cielo.gmontero@gmail.com (C.G.-M.); david.diaz@uah.es (D.D.); lamidolado@gmail.com (E.L.-D.); 2 Ramón y Cajal Institute of Sanitary Research (IRYCIS), 28034 Madrid, Spain; 3 Service of Rehabilitation, National Hospital for Paraplegic Patients, Carr. de la Peraleda, S/N, 45004 Toledo, Spain; mdatienzap@sescam.jccm.es; 4 Immune System Diseases-Rheumatology Service and Internal Medicine, Prince of Asturias University Hospital (CIBEREHD), 28806 Alcala de Henares, Spain

**Keywords:** chronic spinal cord injury (SCI), T cells, CCR6, CD28, immunocompromised

## Abstract

Spinal cord injury (SCI) is a serious medical condition associated with severe morbidities and disability. Chronic SCI patients present an enhanced susceptibility to infections and comorbidities with inflammatory pathogenesis. Chronic SCI appears to be associated with a systemic dysfunction of the immune system. We investigated the alteration of the pivotal CD4+ and CD8+ T lymphocytes in patients with chronic SCI at different years of evolution. A clinically homogenous population of 105 patients with chronic SCI (31 with time of evolution less than 5 years (SCI SP); 32 early chronic (SCI ECP) with time of evolution between 5 and 15 years; and 42 late chronic (SCI LCP) with time of evolution more than 15 years) and 38 healthy controls were enrolled. SCI ECP and SCI LCP patients showed significant CD4+ and CD8+ T lymphopenia, ascribed to a reduction in naïve and CM subsets. Furthermore, SCI ECP and SCI LCP patients showed a significant reduction in the expression of CD28 on CD8+ T lymphocytes. The expression of CCR6 by CD4+ T lymphocytes was decreased during the evolution of chronic SCI, but on CD8+ T lymphocytes, it was observed during the first 15 years of evolution. In conclusion, the chronic SCI course with severe damage to T lymphocytes mainly worsens over the years of disease evolution.

## 1. Introduction

Spinal cord injury (SCI) is a devastating medical condition frequently associated with serious morbidity and disability [1]. Unfortunately, the incidence of SCIs has grown in recent years, especially in men, imposing an important socioeconomic burden on the individual and healthcare systems [2]. Fortunately, advances in clinical healthcare have allowed for an increase in the survival of patients with SCI [3]. However, this achievement has highlighted that the health condition of chronic SCI severely impacts various organic and mental aspects of the patient’s health [4]. Thus, it has been observed that, in addition to recurrent episodes of infection, patients with chronic SCI exhibit a progressive incidence of metabolic, cardiovascular, and mental health disorders [5]. Furthermore, there is evidence of the dynamic nature of neurological injury in SCI, involving progressive cellular degenerative and inflammatory pathological processes that contribute to the potential amplification of tissue damage [6,7]. The identification of the involved pathophysiological mechanisms is highly relevant in order to establish effective prevention of these systemic processes and multiorgan damages.

The immune system is a pivotal defense mechanism against infections, and its deficiency is associated with a predisposition to recurrent and severe infectious events [8,9]. However, the immune system also participates in aberrant responses leading to systemic inflammatory damage and organ dysfunction [10]. There is progressive evidence that an abnormal immune-inflammatory system may play a significant role in the pathogenesis of these systemic complications in patients with chronic SCI [5,11,12]. The impairment of the immune system in patients during the acute phase in the initial weeks or months following SCI is well established [13,14]. However, the potential impact of the immune system on patients with chronic and long-standing SCI remains partially understood [4,10]. SCI appears to be associated with dysfunction of the immune system, a systemic low-grade inflammatory state, and compromised immune cells [12,15].

Among the different cellular populations involved in the immune-inflammatory response, T lymphocytes play a pivotal role both in its regulation and in its effector phase [16]. T lymphocytes are a heterogeneous cellular population, and the two major subsets are CD4 and CD8 lymphocytes, which are referred to as helper and cytotoxic, respectively. Based on the history of antigenic exposure and the phenotypic pattern of CCR7, CD27, or CD62 L expression, different stages of activation/differentiation in T lymphocytes are identified, known as naive (N) and memory, prior to and after antigen exposition. Within the memory stage, distinguished by their tissue migration capacity and functional state, the classifications include central memory (CM), effector memory (EM), and effector (E) T lymphocytes [12]. The expression of other molecules on the T lymphocyte membrane also allows for assessing their activation status and migratory capacity, such as the co-receptor CD28 and the chemoreceptor CCR6, respectively [17,18]. Progressively, the existence of alterations in the T lymphocyte compartment of patients with chronic SCI is being observed [19,20,21]. However, there are conflicting data regarding the distribution and activation of their CD4 and CD8 lymphocyte populations, which may be related to the limited number of patients included in the studies, the restricted and short evolutionary periods encompassed in the analyses, and the clinical heterogeneity of the patients. Further efforts are needed to disentangle the distribution of T lymphocytes in SCI and their potential evolution over the years of disease progression.

Therefore, we aimed to study the subset distribution and activation/differentiation stage of circulating T lymphocytes along the years of evolution of chronic SCI. We investigated a clinically homogeneous population of 105 chronic SCI patients without comorbidities with potential interactions with the immune system and compared them with 38 age- and sex-matched healthy controls (HC). 

## 2. Results

### 2.1. Demographic Profile of Chronic SCI Patients

In Table 1, the main features of the 105 chronic SCI patients and 38 HC included are summarized. Approximately 74.28% of the patients were men, and the mean age was 36.33 ± 13.24 years old. Similarly, 62.10% of the HC were men, and 37.90% were women, with a mean age of 31.41 ± 7.99 years. The mean time of SCI onset was 13.24 ± 9.47 years, a time of evolution.

The neurological level of spinal damage was located within C1–C4, C5–C8, T1–T6, T7–T12, and the lumbosacral metamers in 23.8%, 20%, 26.27%, 20.95%, and 8.57% of patients, respectively. In other words, more than 70.40% of our patients had an SCI above T6. With respect to the ASIA, 46.67% of the patients were AIS A, 16.19% of the patients were AIS B, 16.19% of the patients were AIS C, and 20.95% of the patients were AIS D, indicating that despite 79.04% of the patients exhibiting incomplete lesions, just 62.85% reported incomplete motor injuries, with different extents of intralesional motor preservation and theoretically better mobility profiles.

Medical information from the SCI patients was analyzed in a routine clinical examination in the Physical Medicine and Rehabilitation Department, collecting the following data: (1) baseline demographic features; (2) time and mechanism of injury; (3) neurologic injury level and related severity; (4) tonic and phasic spasticity; (5) presence/absence of pain, its type and seriousness; (6) history of past infections and other indicators of a chronic SCI complication; (7) comorbidities; (8) contemporary medications; (9) fatigue; (10) anxiety and depression levels; (11) degree of independence in daily living activities; and (12) self-reported quality of life and health status.

### 2.2. Patients with Chronic SCI Show a Long-Term Decrease in Circulating CD4+ T Lymphocytes, Explained by a Reduction in the Naïve and CM Subsets

**Figure 1:**
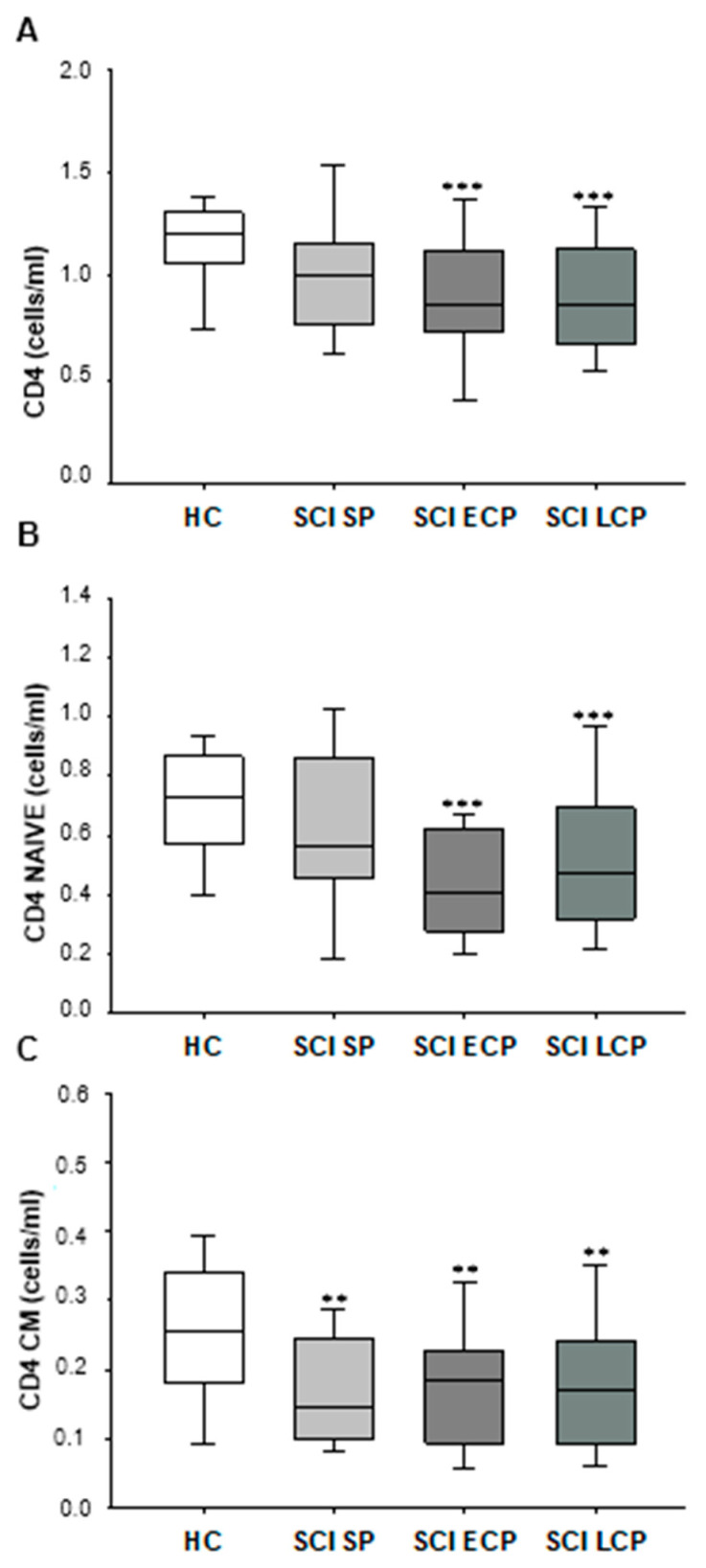
Graphic representation of total CD4+ cells (**A**), CD4+ naïve cells (**B**), and CD4+ central memory (CM) cells (**C**) in patients with SCI SP, SCI ECP, and SCI LCP. *p* < 0.01 (**), *p* < 0.001 (***). SCI SP = Spinal cord injury with a time of evolution less than 5 years. SCI ECP = Spinal cord injury with early chronic with a time of evolution between 5 and 15 years. SCI LCP = Spinal cord injury with long chronicity and a time of evolution greater than 15 years.

First, we studied the absolute number of circulating CD4+ T lymphocytes in chronic SCI patients classified according to the years of follow-up after acute SCI and in HC (Figure 1A) and observed that the SCI SP showed normal counts of circulating CD4 T lymphocytes. However, a significant decrease in the number of circulating CD4+ T lymphocytes in SCI ECP patients was observed compared to the HC group (HC = 1.202 [1.063–1.308] cells/mL, SCI ECP = 0.860 [0.732–1.129] cells/mL, *** *p* = 0.001). This CD4+ lymphopenia was equally marked in patients with SCI LCP (SCI LCP = 0.859 [0.671–1.108] cells/mL, *** *p* = 0.001).

Furthermore, we investigated the counts of the different stages of activation/differentiation of CD4+ T lymphocytes in chronic SCI patients and HC. We found that there was a significant decrease in naïve CD4+ T lymphocytes in patients with SCI ECP compared to HC (HC = 0.727 [0.570–0.0.866] cells/mL, SCI ECP = 0.408 [0.274–0.622] cells/mL, *** *p* = 0.007) (Figure 1B). Significant CD4+ lymphopenia was also quantified in patients with SCI LCP (SCI LCP = 0.527 [0.473–0.322] cells/mL, *** *p* = 0.000) (Figure 1C). Interestingly, we also observed a reduction in the counts of CM CD4+ T lymphocytes in the three groups of SCI patients (HC = 0.256 [0.183–0.337] cells/mL, SCI SP = 0.146 [0.100–0.245] cells/mL, ** *p* = 0.012, SCI ECP = 0.183 [0.094–0.226] cells/mL, ** *p* = 0.014, SCI LCP = 0.170 [0.093–0.241] cells/mL, ** *p* = 0.019). The results of other CD4+ T lymphocyte subsets are shown in the Supplementary Materials.

### 2.3. Patients with Chronic SCI Have a Long-Term Diminution of Circulating CD8+ T Lymphocytes with Early Reduction of the Naïve Subset

**Figure 2:**
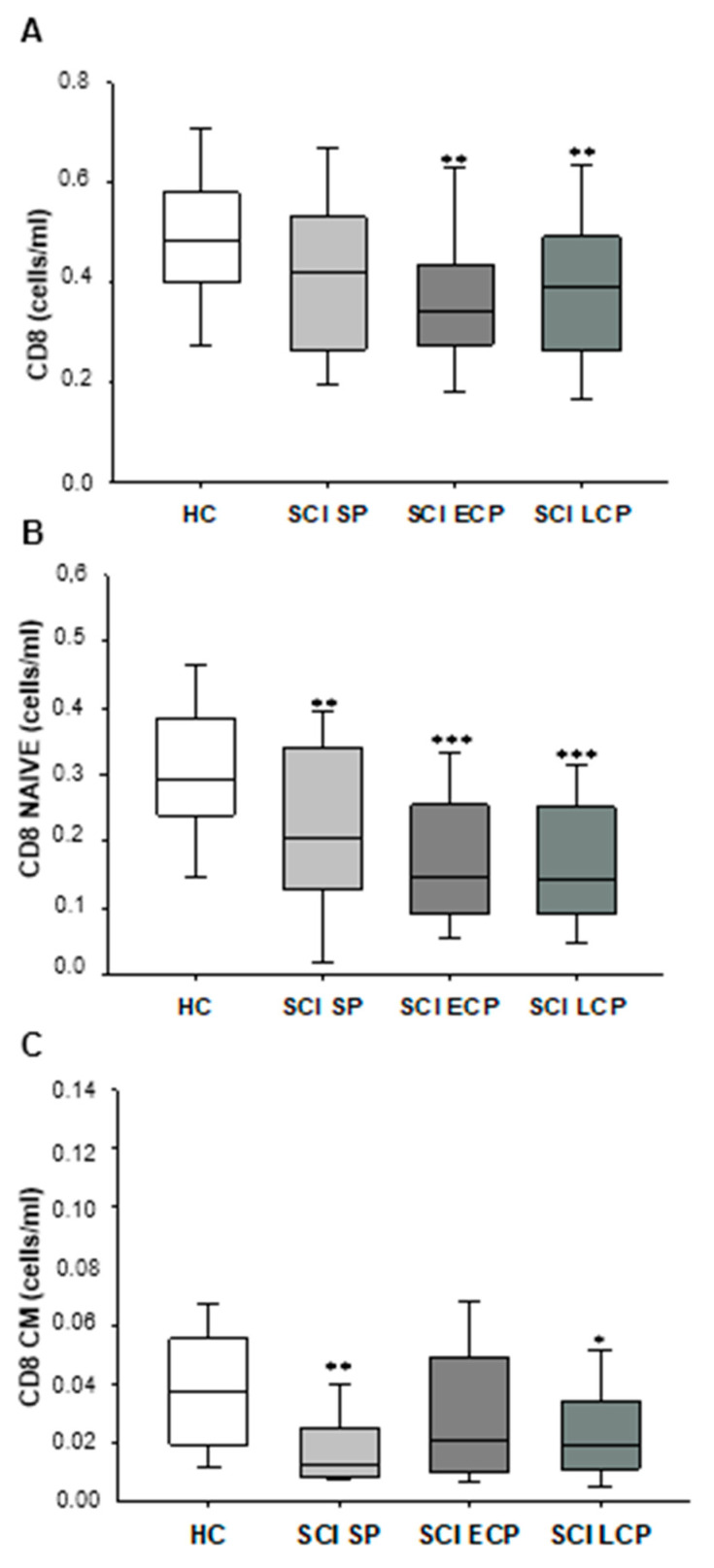
Graphic representation of total CD8+ cells (**A**), CD8 naïve cells (**B**), and CD8+ central memory (CM) cells (**C**) in patients with SCI SP, SCI ECP, and SCI LP. *p* < 0.05 (*), *p* < 0.01 (**), *p* < 0.001 (***). SCI SP = Spinal cord injury with a time of evolution less than 5 years. SCI-ECP = Spinal cord injury with early chronic evolution between 5 and 15 years. SCI LCP = Spinal cord injury with low chronicity with a time of evolution greater than 15 years.

Next, we investigated the counts of circulating CD8+ T lymphocytes in chronic SCI patients, classified according to the years of follow-up after acute SCI and in HC. Our results showed a decrease in the counts of CD8+ T lymphocytes in patients with SCI ECP compared to HC (HC = 0.481 [0.409–0.564] cells/mL, SCI ECP = 0.342 [0.275–0.434] cells/mL, * *p* = 0.023). In addition, patients with SCI LCP also showed a significant reduction in circulating CD8+ T lymphocytes (SCI LCP = 0.391 [0.276–0.490] cells/mL, ** *p* = 0.012) (Figure 2A).

We also quantified the counts of the different stages of activation/differentiation of CD8+ T lymphocytes in the different groups of chronic SCI patients and HC. We observed a significant decrease in naïve CD8+ T lymphocytes in patients with SCI SP with respect to HC (HC = 0.292 [0.234–0.373] cells/mL, SCI SP = 0.206 [0.127–0.340] cells/mL, *** *p* = 0.005). A decrease in naïve CD8+ T lymphocytes was found in the three groups of SCI patients with respect to HC and was more marked in patients with SCI ECP and SCI LCP (SCI ECP = 0.146 [0.090–0.254] cells/mL, *** *p* = 0.000; SCI LCP = 0.143 [0.093–0.252] cells/mL, *** *p* = 0.000) (Figure 2B). We also observed a significant decrease in CM CD8+ T lymphocytes in patients with SCI SP and SCI LCP compared to HC (HC = 0.037 [0.198–0.055] cells/mL, SCI SP = 0.013 [0.008–0.025] cells/mL, ** *p* = 0.004; SCI LCP = 0.019 [0.011–0.032] cells/mL, * *p* = 0.011). (Figure 2C). The results of other CD8+ T lymphocyte subsets are shown in the Supplementary Materials.

### 2.4. Chronic SCI Patients Show Maintained Reduced Expression of CD28+ on CD8+ T Lymphocytes and on CD4+ T Lymphocytes Selectively in Those with Long-Term Evolution

**Figure 3:**
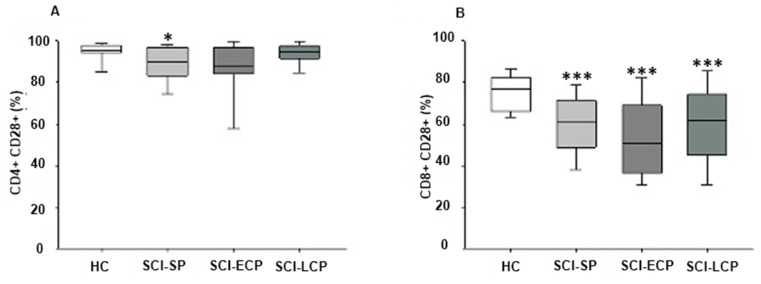
Percentage of CD4+ CD28+ cells (**A**) and CD8+ CD28+ cells (**B**) in patients with SCI SP, SCI ECP, and SCI LCP. *p* < 0.05 (*), *p* < 0,001 (***). SCI SP = Spinal cord injury with a time of evolution less than 5 years. SCI-ECP = Spinal cord injury with early chronic with a time of evolution between 5 and 15 years. SCI LCP = Spinal cord injury with low chronicity with a time of evolution greater than 15 years.

We investigated the expression of the coreceptor CD28+ on CD4+ and CD8+ T lymphocytes from the different groups of chronic SCI classified according to the years of evolution after the injury. We found a decrease in the percentage of CD4+ T lymphocytes expressing CD28 in patients with SCI SP with respect to HC (HC = 95.2 [94.2–97.4] %, SCI SP = 90.1 [84.5–96.7] %, * *p* = 0.024) (Figure 3A).

We studied the expression of CD28 on CD8+ T lymphocytes in chronic SCI patients and HC. We found a reduction in the percentage of CD8+CD28+ T lymphocytes in the three groups of chronic SCI patients with respect to HC. The percentage of CD8+ T lymphocytes expressing CD28+ was significantly reduced in patients with SCI SP compared to HC (HC = 77 [66–79.5] %, SCI SP = 61 [49.5–70.9] %, *** *p* = 0.000). Likewise, a similar decrease in the percentage of these cells was observed in SCI ECP and LCP (SCI ECP = 50.5 [36.3–69] %, SCI LCP = 61.85 [46.3–73.9] % *** *p* = 0.000) (Figure 3B).

### 2.5. Patients with Spinal Cord Injury Have a Decreased Percentage and Number of Chemokine Receptor CCR6+ CD8+ T Lymphocytes and CD4+ T Lymphocytes

**Figure 4:**
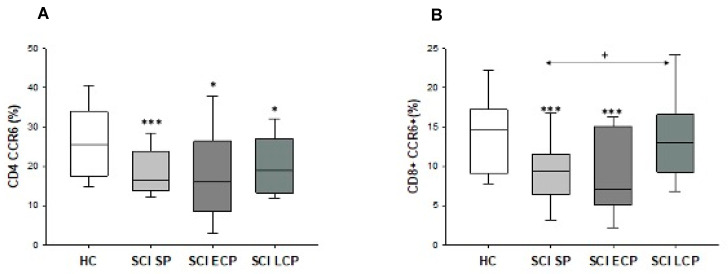
Percentage of CD4+ CCR6+ cells (**A**) and CD8+ CCR6+ cells (**B**) in patients with SCI SP, SCI ECP, and SCI LCP. *p* < 0.05 (*), *p* < 0.001 (***). SCI SP = Spinal cord injury with a time of evolution less than 5 years. SCI-ECP = Spinal cord injury with early chronic with a time of evolution between 5 and 15 years. SCI LCP = Spinal cord injury with low chronicity with a time of evolution greater than 15 years.

Finally, we investigated the expression of CCR6+ on CD4+ and CD8+ T lymphocytes in chronic SCI patients and HC. We observed that the SCI SP patients showed a significant decrease in the percentage of CCR6+CD4+ T lymphocytes compared to the HC group (HC = 25.45 [18–32.1] %, SCI SP = 16.5 [14.25–23.4] %, *** *p* = 0.007). Similarly, a decrease was also found in patients with SCI ECP and LCP (SCI ECP = 17.4 [9.73–26.5] %, SCI LCP = 18.9 [13.2–27] %, * *p* = 0.038 and * *p* = 0.026) (Figure 4A).

On the other hand, our results also showed a decrease in the percentage of CCR6+CD8+ T lymphocyte populations in patients with SCI SP and SCI ECP compared to HC (HC = 14.70 [9.20–17.00] %, SCI SP = 9.37 [6.52–11.50] %, *** *p* = 0.008, SCI ECP = 7.09 [5.16–15.10] %, *** *p* = 0.005) (Figure 4B).

## 3. Discussion

In this study, we demonstrated that patients with chronic SCI without comorbidities show severe and progressive damage to the circulating T lymphocyte compartment, with a reduction in CD4+ and CD8+ T lymphocyte subsets that worsen after 5 years of evolution. This CD4+ and CD8+ T lymphocyte shrinking is mainly ascribed to the naïve and CM activation/differentiation stages. Furthermore, CD8+ T lymphocytes from these chronic SCI patients showed an early and maintained reduction in the expression of CD28, while CD4+ T lymphocytes showed a reduction only in those in the first 5 years of evolution. T lymphocytes of chronic SCI patients also show a reduction in the percentage of those cells expressing CD28 and an increase in the expression of CCR6.

T lymphocytes play a central role in the immune response [22]. CD4+ T lymphocytes are critical cells in the regulation of the innate and adaptative immune response, and CD8+ T lymphocytes are essential effectors of the adaptative response. Abnormalities in both T lymphocyte populations have been involved in the pathogenesis of different chronic inflammatory diseases as well as in the defective response to infections [23]. CD4+ and CD8+ T lymphocytes are also major players in SCI pathogenesis. It is well-described that T lymphocytes are recruited in the injured spinal cord and interact with the different parenchymal cells, such as macrophages/microglia, oligodendrocytes, astrocytes, and others, modulating their behavior, activation, and proliferation, thus affecting the clinical progression of SCI [24]. In more detail, both CD4 and CD8+ lymphocytes have direct effects on neutrophilic inflammation, disruption of the blood-spinal cord barrier (BSCB), remyelination, and neuropathic pain following SCI [25,26]. Therefore, the role of CD4 and CD8 T lymphocytes in the early stages of SCI and their relevance in SCI progression are well documented, although little is known about their status and implications in the chronic stages. 

There is experimental evidence that chronic SCI is associated with systemic alterations of cellular and molecular components of the immune system, with marked involvement of T lymphocytes [19,20,21]. In addition, alterations in circulating immune cells have also been observed in patients with chronic SCI. However, the pattern of evolution of immune dysfunction over the years of follow-up has not been established. In our study of 105 patients with chronic SCI, we observed that the temporal evolution of the disease was associated with a significant reduction in the counts of circulating CD4+ and CD8+ T lymphocytes that appeared after 5 months of follow-up. These results agree with previous findings of reduced absolute counts of both subsets of circulating T lymphocytes [10,13,27,28]. CD4+ and CD8+ T lymphocytes are heterogeneous populations with different stages of activation/differentiation [29]. Our findings show a nonhomogeneous involvement of the different subpopulations of CD4+ and CD8 +T lymphocytes in patients with chronic SCI. In both circulating T lymphocyte populations, the reduction observed in SCI patients can be attributed to the naïve and CM subsets. The cause of this severe CD4+ and CD8+ deficiency remains unknown; however, different mechanisms that are not mutually exclusive may be involved. First, it seems that patients with chronic SCI present a long-term functional limitation of the bone marrow that may be involved in the demonstrated progressive CD4+ and CD8+ T lymphopenia found in these subjects. Second, our results indicate a severe reduction in the number of circulating naïve CD4+ and CD8+ T lymphocytes in patients with chronic SCI. It is known that thymus output plays a critical role in generating naïve T lymphocytes [10]. To our knowledge, the present study constitutes the first evidence of defective production of naïve T cells in chronic SCI. We thus hypothesize that the thymus of patients with chronic SCI suffers an involution ligated to the different local and systemic mechanisms ligated to the progression of this condition [4,11,28], aiding to explain the reduced production of naïve T cells in these patients. Third, the simultaneous observed reduction in the naïve and CM and normal counts of EM and E activation/differentiation stages of CD4+ and CD8+ T lymphocytes indicate a bias in the T lymphocyte subset distribution in these patients. A dynamic of evolution of naïve T lymphocytes toward memory cells and from these toward E and EM stages has been established [22]. Thus, it is possible to suggest that chronic SCI is associated with an antigen and/or cytokine environment favoring the final stages of activation/differentiation of T lymphocytes. The potential autoimmunity and microorganism pressure suffered by these patients may play a role in this immune system dysfunction. Fourth, the potential anomalous tissue distribution of CD4 and CD8 T lymphocytes in chronic SCI patients can also be involved in the observed lymphopenia. In this sense, T-cell infiltration of the spinal cord has been shown in patients with SCI [7]. Furthermore, experimental models and patients with chronic SCI suffer gut dysbiosis, intestinal barrier damage, and recurrent or chronic bacterial urinary infections with potential local activation of T lymphocytes [30]. It may be possible to claim that diseases associated with chronic SCI with potential damage to the immune system could be the cause of the demonstrated persistent subset redistribution of circulating CD4+ and CD8+ T lymphocytes. However, the stringent exclusion criteria used in the inclusion of the patients in our study eliminated this possibility. Although recent urinary or other acute bacterial infections were exclusion criteria, a history of urinary infections might play a role in T lymphocyte deficiency, as previously indicated. Furthermore, the distribution of HC and similar epidemiological origins also support that the CD4+ and CD8+ T lymphocyte abnormalities found in patients are due to chronic SCI [31], although other variables like age can also play a role in these differences. The relevance of the involvement of T lymphocytes in patients with chronic SCI is also supported by previous findings. Functional disturbance of CD4+ T lymphocytes with expansion of activated regulatory CD4+ lymphocytes and Th17 polarized lymphocytes has been described [27]. In this line, we recently described how the temporal evolution of chronic SCI affects specific Treg subsets. In more detail, we reported that SCI-ECP and SCI-LCP patients seemed to present increased proportions of CD4+ CD25+/low Foxp3+ Tregs in comparison to HC, whereas a decreased number of these cells expressing CCR5 was observed in SCI-SP, SCI-ECP, and SCI-LCP patients. Furthermore, an increased number of CD4+ CD25+/high/low Foxp3 with negative expression of CD45RA and CCR7 was observed in SCI-LCP patients when compared to the SCI-ECP group [20]. Contradictory results have been shown about the potential activation of interferon-gamma-polarized CD4+ lymphocytes in chronic SCI [32]. The observed dependency of the T lymphocyte abnormalities at the time of evolution of the chronic SCI might shed some light on the understanding of these discrepancies.

In this study, we also studied the expression of CD28 by CD4+ and CD8+ T lymphocytes from chronic SCI. CD28 is a primary costimulatory molecule for T-cell activation, leading to the induction of several transcriptional activators and molecular pathways [33]. The reduction in the expression of CD28 by T lymphocytes has been associated with a chronic state of immune system activation in rheumatoid arthritis [34]. Furthermore, the downregulation of CD28 expression by T lymphocytes has been associated with aging as well as with different chronic immune-based diseases [35,36]. An increase in CD28-T lymphocytes also appears to be associated with the presence of chronic infections and can be a prognostic indicator [37]. Our results showed a transient early expansion of CD28-CD4 T lymphocytes in chronic SCI patients during the first 5 years after acute injury. However, the expansion of CD28-CD8 T lymphocytes is detectable early and is maintained during the temporal evolution of chronic SCI patients. These findings support a persistent state of activation of CD8+ T lymphocytes in chronic SCI patients. The expansion of CD28-CD8+ T lymphocytes may support a stage of energy-deficient activation of these pivotal effector immune cells. Our results are also in agreement with previous studies reporting premature senescence of the IS in chronic SCI [38,39,40].

Finally, chemokines and their receptors have been largely studied in SCI, playing a multifunctional role in distinct cellular cascades, depending on their induction time point and anatomical localization [41]. Some chemokines are dysregulated in chronic SCI [42]. Likewise, abnormal patterns of chemokine receptors by immune system cells have also been described in patients with chronic SCI [41]. We observed decreased expression of CCR6 by CD4+ and CD8+ T lymphocytes in chronic SCI patients during the evolution of the disease. However, we observed normal expression of CCR in these cells. Interestingly, Monahan et al. [21] observed that patients with chronic SCI showed an increase in the expression of the chemokine CCR4+ only by a subset of CD4 T lymphocytes. CCR6 is a critical receptor activated by C-C motif chemokine ligand 20 (CCL20). Interestingly, the importance of this interaction has been reported in other neuroinflammatory conditions [43]. CCL20 has been previously detected in patients with SCI, with an important regulatory role in Th17 polarization [44]. To our knowledge, this is the first study to identify the involvement of the downregulation of CCR6+ T-cell subsets in patients with chronic SCI. These findings also support the ability of the loss of T cells to orchestrate an appropriate immune response in these patients.

Our study has some important limitations, especially considering the sex and age characteristics of HC and the different chronic SCI subgroups, which can also contribute to the observed differences in CD4 and CD8 T cells. In this sense, previous studies have reported that females present a higher number of circulating CD4 cells [45], whereas the number of CD4 and CD8 cells tends to decline with age [46]. As we classify SCI patients considering the time of evolution since initial injury as a critical variable in our study, it is difficult to adjust the age between those with a short evolution period (1–5 years) and longer (5–15 and more than 15 years). Similarly, according to epidemiological data, males are more likely to suffer from SCI than females [47], explaining the high proportion of males included in our study and the disparities in the proportion of males/females more evident in the SCI-SP group. These differences in sex and age between controls and patients should also be considered in the interpretation of our results, to address them in future studies.

Taken together, our findings demonstrate a marked and persistent involvement of CD4 and CD8 T lymphocyte populations in chronic SCI patients. Our results highlight the state of T-lymphocytic immunodeficiency suffered by these patients, which has worsened over the years. The intensity of these deficiencies of T lymphocytes can be related to the predisposition to suffer from recurrent and severe infections, the poor response to vaccinations, and the development of different immune-mediated diseases, such as those of a metabolic and vascular nature. It can be argued that these alterations could become biomarkers of the systemic involvement of chronic SCI and raise the need to introduce new immune intervention strategies in these patients.

## 4. Materials and Methods

### 4.1. Study Design

A total of 105 patients with chronic SCI were included in this prospective study. The inclusion criteria considered in this study were: (1) being ≥18 years; (2) history of SCI in a period ≥1 year, occurring at any level; and (3) SCI with varied severity, ranging from grades A to E according to the American Spinal Injury Association (ASIA) Impairment Scale (AIS). A Physical and Rehabilitation Medicine board-certified clinician in SCI medicine evaluated the subjects’ injuries according to the International Standards for Neurologic Classification of Spinal Cord Injury [48,49]. Among the exclusion criteria there were: (1) a coincident infection with notable severity, like urinary tract infection (UTI) or a respiratory infection, evidenced with a positive culture in the last 3 months; (2) chronic viral or bacterial infection; (3) clinical diagnosis of an autoimmune disease; (4) serious cardiovascular disease (CVD); (5) hematopoietic, renal, lung or hepatic complications; (6) an endocrine or metabolic disorder, (i.e., type 1/2 diabetes mellitus); (7) Previous history of cancer; (8) pressure ulcers in the last year; (9) administration of immunomodulatory drugs like steroids in the last 3 months; (10) suffering from immunodeficiency or malnutrition; (11) being in pregnancy or lactation period; and (12) having undergone any psychiatric disorder. To accurately address the effect of the SCI time course on IS, the recruited patients were subdivided into three subpopulations: short period (SCI SP), if the time of evolution was less than 5 years; early chronic (SCI ECP), if the time of evolution was between 5 and 15 years; and late chronic (SCI LCP), if the time of evolution was more than 15 years. Concurrently, these patients were compared with 38 HC.

The present work was performed in agreement with the basic ethical principles of autonomy, beneficence, non-maleficence, and distributive justice, following the statements of Good Clinical Practice, the principles contained in the most recent Declaration of Helsinki (2013), and the Oviedo Convention (1997). The data and information collected followed current legislation on data protection (Organic Law 3/2018 of December 5, Protection of Personal Data and Guarantee of Digital Rights and Regulation (EU) 2016/679). All patients were properly informed before enrolment, and a signed written consent approved by the Institutional Review of the National Hospital for Paraplegic Patients (10 September 2015) was obtained from each subject.

Medical information from the SCI patients was analyzed in a routine clinical examination in the Physical Medicine and Rehabilitation Department, collecting the following data: (1) baseline demographic features; (2) time and mechanism of injury; (3) neurologic injury level and related severity; (4) tonic and phasic spasticity; (5) presence or absence of pain, its type and seriousness; (6) history of past infections and other indicators of a chronic SCI complication; (7) comorbidities; (8) contemporary medications; (9) fatigue; (10) anxiety and depression levels; (11) degree of independence in daily living activities; and (12) self-reported quality of life and health status.

Blood samples were extracted from all subjects via standard venipuncture using an established aseptic technique. Samples were obtained from chronic SCI patients at the time of the medical evaluation in the outpatient clinic area.

### 4.2. Isolation of Peripheral Blood Mononuclear Cells

Peripheral blood mononuclear cells (PBMCs) were separated by using Ficoll-Hypaque (LymphoprepTM, Axis-Shield, Oslo, Norway) gradient centrifugation. Subsequently, cells were resuspended in RPMI 1640 (BioWhittaker Products, Verviers, Belgium) supplemented with 10% heat-inactivated fetal calf serum, 25 mM HEPES (BioWhittaker Products), and 1% penicillin-streptomycin (BioWhittaker Products). Cell enumeration was carried out by conventional light microscopy in a Neubauer chamber following the trypan blue dead cell exclusion criteria. The viability of PBMCs was assessed by both trypan blue (light microscopy) and 7-aminoactinomycin D (7-AAD) (flow cytometry) exclusion.

### 4.3. Immunophenotype Studies

The number of T lymphocyte subpopulations was determined in fresh PBMCs by ten-color polychromatic flow cytometry in a FACSAria cytometer using FACSDiva software (Becton Dickinson, Franklin Lakes, NJ, USA). Half a million PBMCs were incubated with a combination of the following monoclonal antibodies: CD95-FITC, CD40 L-PE, CD4-PerCP, CD45RA-APC, CD3-Alexa 700, CD28-c-PE-Cy7, CD27 APC-Alexa 780, and CD8-Alexa 405. For these procedures, CD95-FITC, CD40 L-PE, CD4-PerCP, CD45RA-APC, and CD3-Alexa 700 were obtained from Beckton Dickinson; CD27 APC-Alexa 780 and CD8-Alexa 405 were obtained from Invitrogen (Invitrogen, Carlsbad, CA, USA); and CD28-c-PE-Cy7 was obtained from eBioscience (e-Bioscience, San Diego, CA, USA).

The expression of chemokine receptors on T lymphocyte subsets and subpopulations was determined in half a million fresh PMBCs by eight-color polychromatic flow cytometry in a FACSAria cytometer using FACSDiva software version 9.0 (Becton Dickinson, Franklin Lakes, NJ, USA). Half a million PBMCs were incubated with a combination of monoclonal antibodies: CCR4-c-PE-Cy7, CCR5-FITC, CCR6-PE, CXCR5-PERCEP-Cy-5.5, CD45RA-APC, CD3-Alexa 700, CD27 APC-Alexa 780, and CD4-Alexa 405. For these procedures, CCR4-c-PE-Cy7, CCR5-FITC, CCR6-PE, and CXCR5-PerCP-Cy-5.5 were obtained from Biolegend (Biolegend, San Diego, CA, USA); CD45RA-APC and CD3-Alexa 700 were obtained from Beckton Dickinson; and CD27 APC-Alexa 780 and CD8-Alexa 405 were obtained from Invitrogen (Invitrogen, Carlsbad, CA, USA).

Once the monoclonal antibodies are added, cells are incubated for 20 min at 4 °C in the dark. Afterwards, cells are washed in a PBS solution to eliminate extra antibodies, and 100 µL of PBS is then added for acquisition by flow cytometry. Control studies with unstained cells and cells incubated with isotype-matched irrelevant monoclonal antibodies were included for every experiment. In the forward scatter-side scatter (FSC-SSC) dot plot, a biparametric gate was represented around the lymphocyte population. Analyses were performed using FlowJo software version 10 (TreeStar Inc., Ashland, OR, USA).

### 4.4. Statistical Analysis

Nonparametric Mann–Whitney U tests were applied to compare chronic SCI patients and HC. All calculations were carried out with the Statistical Package for the Social Sciences (SPSS, version 22.0, Chicago, IL, USA). Significance was established at *p*-values (*p*) *p* < 0.05 (*), *p* < 0.01 (**), and *p* < 0.001 (***).

## 5. Conclusions

In this study, we observed that patients with chronic SCI present an abnormal T-cell distribution and count in comparison to healthy controls. More specifically, a decrease in absolute T cells, CD4+ and CD8+, as well as their naïve and CM populations, can be reported in these patients. In addition, the diminished levels of CCR6 and CD28 can be related to the immunocompromised status of these patients, thereby explaining the high risk of suffering from infections and other diseases with inflammatory pathogenesis.

## Figures and Tables

**Table 1:** Demographic data of healthy controls (HC) and patients with SCI SP, SCI ECP, and SCI LP SCI SP = Spinal cord injury with a time of evolution less than 5 years. SCI-ECP = Spinal cord injury with early chronic with a time of evolution between 5 and 15 years. SCI LCP = Spinal cord injury with low chronicity with a time of evolution greater than 15 years.

Variable	HC (*n* = 38)	SCI (*n* = 105)	SCI-SP (*n* = 31)	SCI-ECP (*n* = 32)	SCI-LCP (*n* = 42)
Age (years)	31.41 ± 7.99	36.33 ± 13.24	29.24 ± 14.40	36.81 ± 12.26	40.28 ± 17.65
Sex (men/ women)	62.10%/ 37.90%	74.28%/25.72%	90.32%/9.68%	77.78%/22.22%	61.70%/ 38.30%
Time of injury(years)		13.24 ± 9.47	2.30 ± 1.54	10.11 ± 2.55	22.26 ± 5.33
ASIA					
A		46.67%	41.93%	55.55%	44.68%
B		16.19%	3.70%	3.70%	21.28%
C		16.19%	18.51%	18.52%	19.15%
D		20.95%	22.22%	22.22%	14.89%
Injury level					
C1–C4		23.80%	22.22%	22.22%	14.89%
C5–C8		20.00%	18.51%	18.52%	25.53%
T1–T6		26.27%	25.92%	25.93%	29.79%
T7–T12		20.95%	29.62%	29.63%	14.89%
L1–L6		8.57%	3.70%	3.70%	14.89%

## Data Availability

The data used to support the findings of the present study are available from the corresponding author upon request.

## Supplementary Materials

The following supporting information can be downloaded at: https://www.mdpi.com/article/10.3390/ijms242417596/s1.
