# Supplementary material for: Patients with Chronic Spinal Cord Injury Display a Progressive Alteration over the Years of the Activation Stages of the T Lymphocyte Compartment
Source: Int J Mol Sci. 2023 Dec 18;24(24):17596. doi: 10.3390/ijms242417596 (PMC10744286; doi:10.3390/ijms242417596)
Supplement: Supplementary file 1 [file ijms-24-17596-s001.zip › ijms-2744534-supplementary.pdf]

Supplementary material

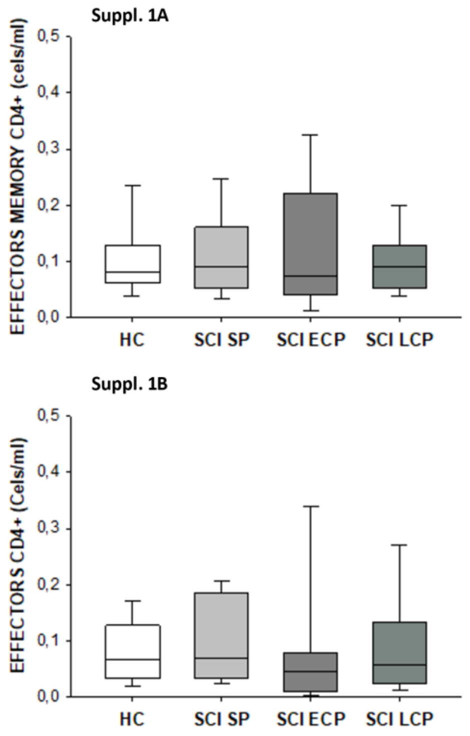

Suppl. S1A-B. Number of effector and effector memory CD8 in patients with chronic SCI SP, SCI ECP and SCI LP in comparison to HC.

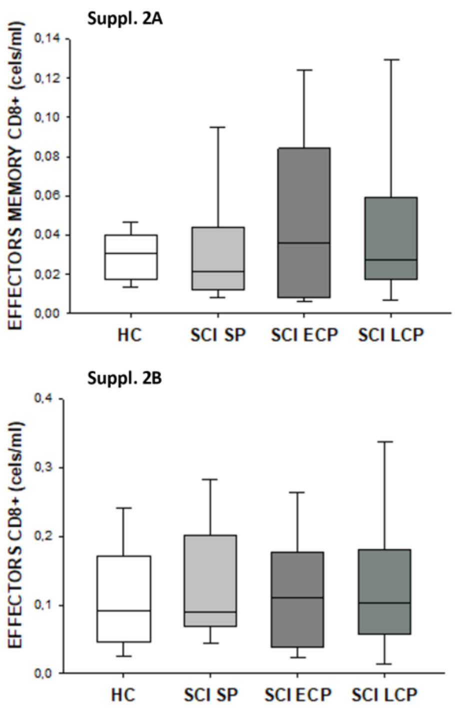

Suppl. S2A-B. Number of effector and effector memory CD8 in patients with chronic SCI SP, SCI ECP and SCI LP in comparison to HC.

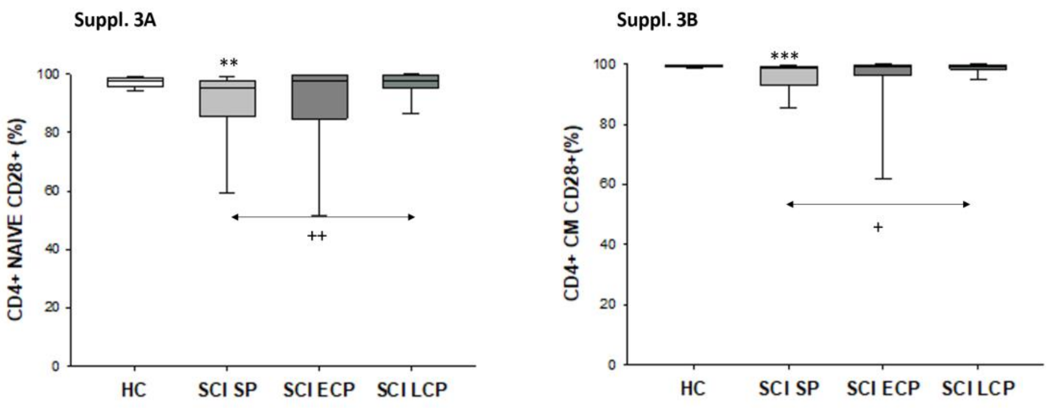

Suppl. S3A-B. Percentage of CD4 CD28 naïve (A) and central memory (B) cells in patients with chronic SCI SP, SCI ECP and SCI LP in comparison to HC. p<0.05 (*), p <0.01 (**), p <0.001 (***)

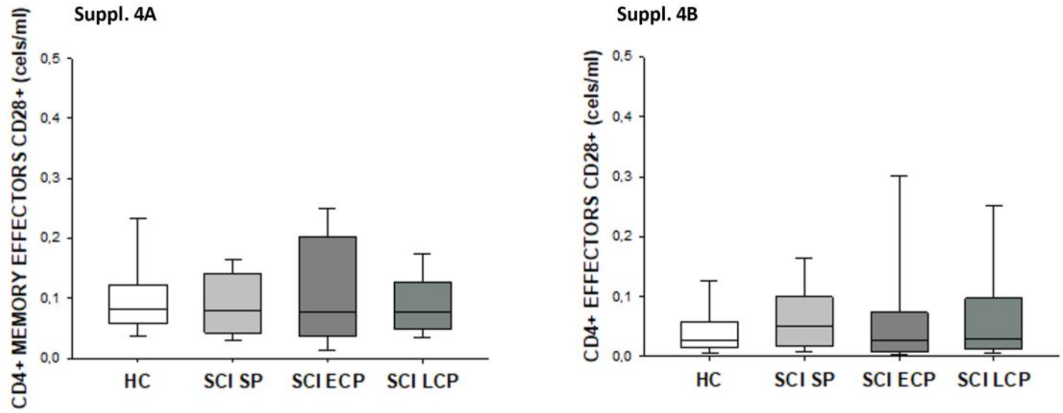

Suppl. S4A-B. Number of CD4 CD28 memory effectors (A) and effectors (B) cells in patients with chronic SCI SP, SCI ECP and SCI LP in comparison to HC.

Suppl. 5A

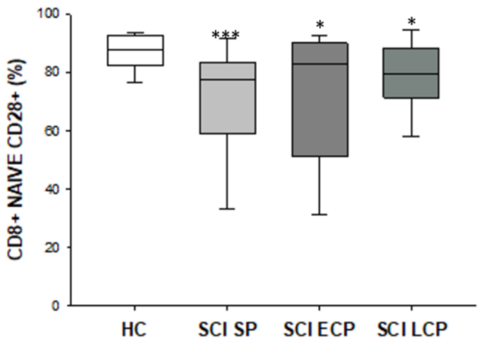

Suppl. 5B

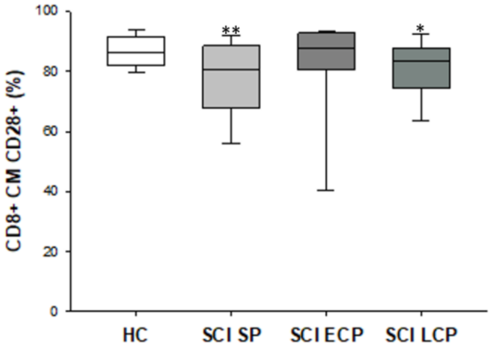

Suppl. S5A-B. Percentage of CD8 CD28 naïve (A) and central memory (B) cells in patients with chronic SCI SP, SCI ECP and SCI LP in comparison to HC. $p<0.05$ (*), $p<0.01$ (**), $p<0.001$ (***)

Suppl. 6A

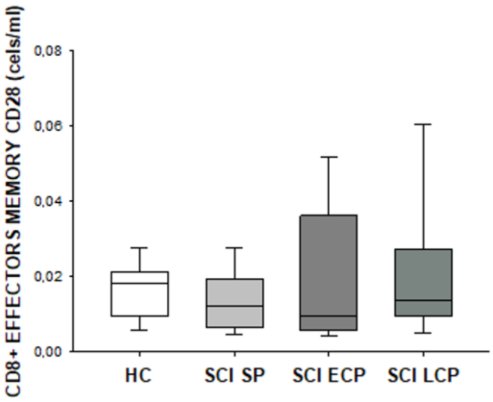

Suppl. 6B

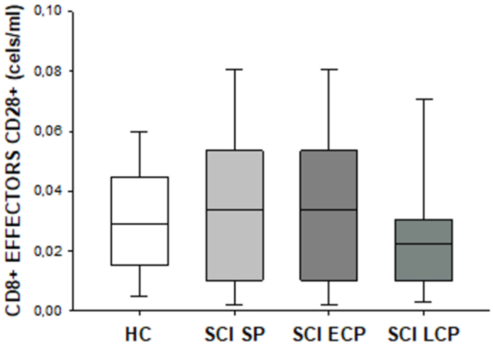

Suppl. S6A-B. Number of CD8 CD28 memory effectors (A) and effectors (B) cells in patients with chronic SCI SP, SCI ECP and SCI LP in comparison to HC.

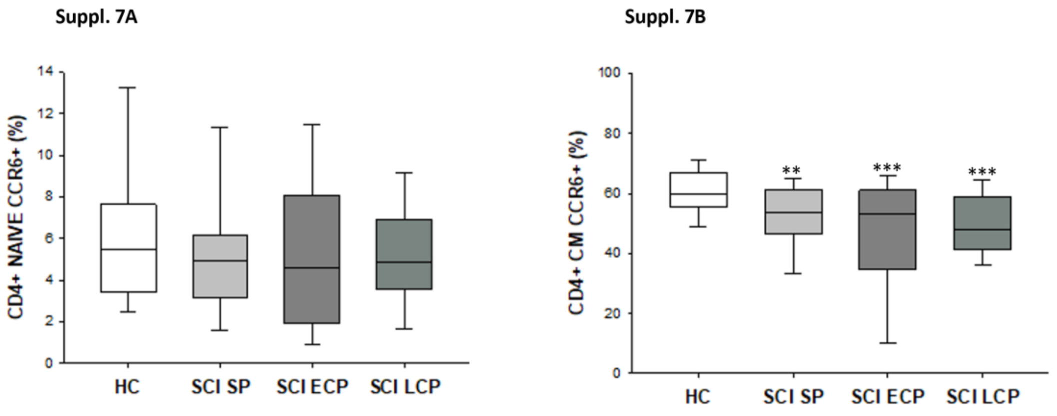

Suppl. S7A-B. Percentage of CD4 CCR6 naïve (A) and central memory (B) cells in patients with chronic SCI SP, SCI ECP and SCI LP in comparison to HC. p<0.05 (*), p <0.01 (**), p <0.001 (***)

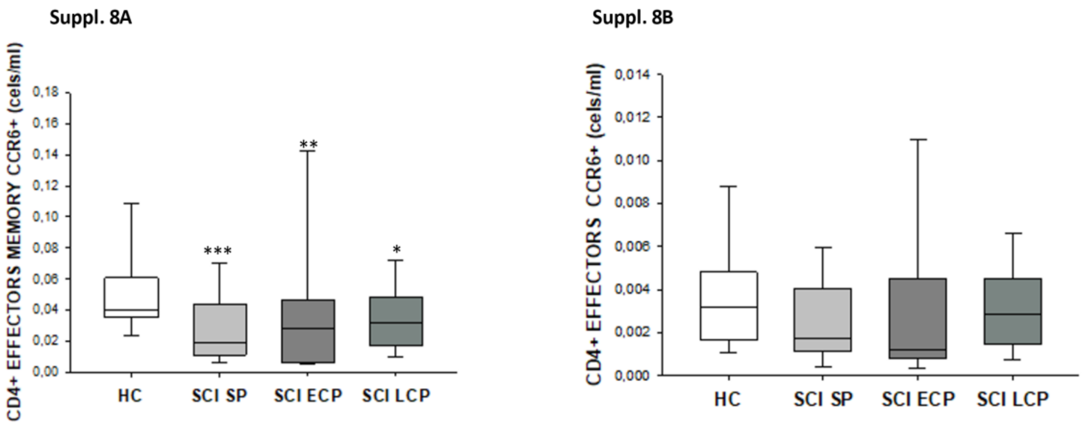

Suppl. S8A-B. Number of CD4 CCR6 memory effectors (A) and effectors (B) cells in patients with chronic SCI SP, SCI ECP and SCI LP in comparison to HC. p<0.05 (*), p <0.01 (**), p <0,001 (***)

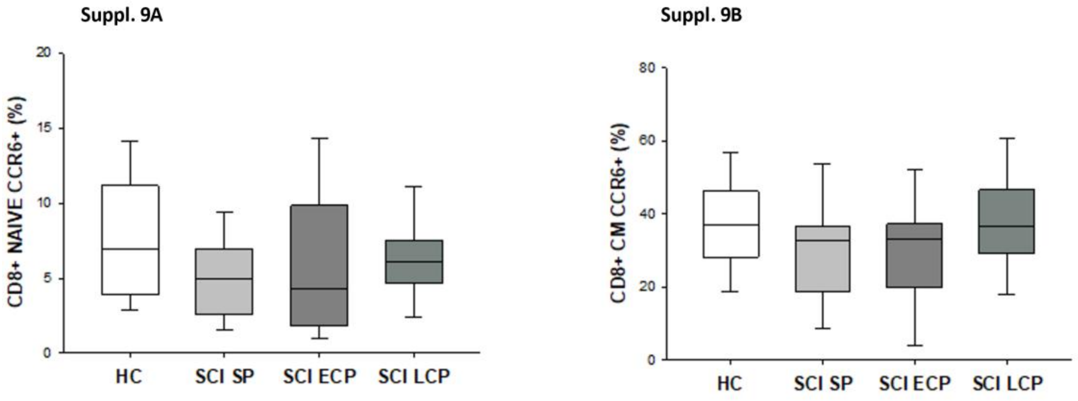

Suppl. S9A-B. Percentage of CD8 CCR6 naïve (A) and central memory (B) cells in patients with chronic SCI SP, SCI ECP and SCI LP in comparison to HC

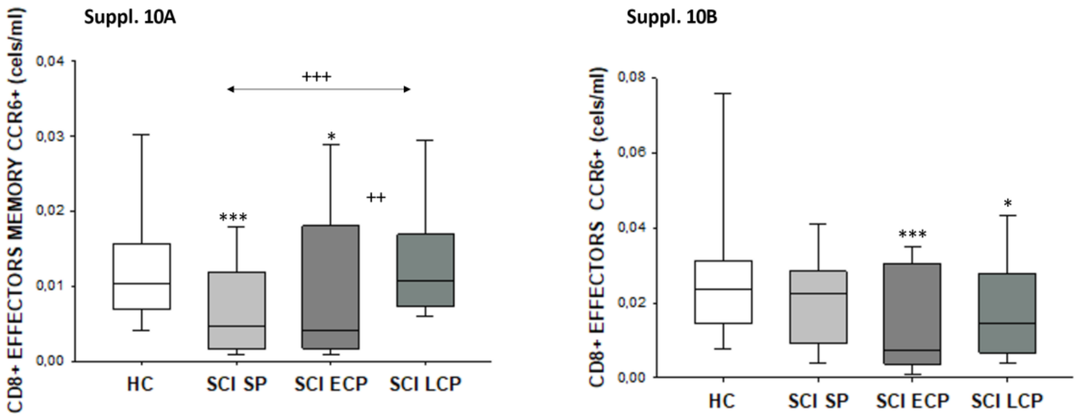

Suppl. S10A-B. Number of CD8 CCR6 memory effector (A) and effector (B) cells in patients with chronic SCI SP, SCI ECP and SCI LP in comparison to HC . $p<0.05$ (*), $p<0.01$ (**), $p<0.001$ (***).
